# Data-driven approach for creating synthetic electronic medical records

**DOI:** 10.1186/1472-6947-10-59

**Published:** 2010-10-14

**Authors:** Anna L Buczak, Steven Babin, Linda Moniz

**Affiliations:** 1 Johns Hopkins University Applied Physics Laboratory, 11100 Johns Hopkins Rd, Laurel, MD 20723-6099, USA

## Abstract

**Background:**

New algorithms for disease outbreak detection are being developed to take advantage of full electronic medical records (EMRs) that contain a wealth of patient information. However, due to privacy concerns, even anonymized EMRs cannot be shared among researchers, resulting in great difficulty in comparing the effectiveness of these algorithms. To bridge the gap between novel bio-surveillance algorithms operating on full EMRs and the lack of non-identifiable EMR data, a method for generating complete and synthetic EMRs was developed.

**Methods:**

This paper describes a novel methodology for generating complete synthetic EMRs both for an outbreak illness of interest (tularemia) and for background records. The method developed has three major steps: 1) synthetic patient identity and basic information generation; 2) identification of care patterns that the synthetic patients would receive based on the information present in real EMR data for similar health problems; 3) adaptation of these care patterns to the synthetic patient population.

**Results:**

We generated EMRs, including visit records, clinical activity, laboratory orders/results and radiology orders/results for 203 synthetic tularemia outbreak patients. Validation of the records by a medical expert revealed problems in 19% of the records; these were subsequently corrected. We also generated background EMRs for over 3000 patients in the 4-11 yr age group. Validation of those records by a medical expert revealed problems in fewer than 3% of these background patient EMRs and the errors were subsequently rectified.

**Conclusions:**

A data-driven method was developed for generating fully synthetic EMRs. The method is general and can be applied to any data set that has similar data elements (such as laboratory and radiology orders and results, clinical activity, prescription orders). The pilot synthetic outbreak records were for tularemia but our approach may be adapted to other infectious diseases. The pilot synthetic background records were in the 4-11 year old age group. The adaptations that must be made to the algorithms to produce synthetic background EMRs for other age groups are indicated.

## I. Background

### I.a Motivation

Despite the current push to adopt electronic medical records (EMRs) as the standard for patient records, research concerned with utilizing all the information in EMRs may be compromised because legal restrictions and privacy concerns limit access to EMRs in academic and industrial research settings to a small number of institutions that have access to the full records. For example, any algorithm designed to work on EMRs can only be tested on a specific set of records so that there is no consistent set of test data that can be used by all interested parties to compare the efficacy of different algorithms. To avoid compromising patient privacy and hospital proprietary concerns, the intent of the Synthetic Electronic Medical Records Generator (EMERGE) project is to develop a methodology for creating synthetic EMRs from a set of real EMRs. Using EMERGE, test beds could be synthesized, creating EMRs for both background records and artificial outbreaks or emergencies that are not present in the real data. The availability of a standardized set of test data would allow comparison of different algorithms and procedures that operate on EMRs as well as provide a set of records for the development of such algorithms.

There are privacy and proprietary concerns with the dissemination of medical record data [1]. These concerns remain even if it is sanitized or anonymized [2,3] because of the variety of different types and sources of data tied together within the EMR. Even having just the compound of ZIP code, gender, and date of birth has been described as being sufficient for uniquely identifying a large percentage of the population of the United States [4]. The development of synthetic EMR data for both background records and injected disease records is thus vital for increased research in the mining of data in EMRs to improve quality of care, detection of health threats, and monitoring for adverse drug effects.

The first EMERGE product provided us with valuable lessons that were used in the methodology described in this paper. This earlier product was a set of synthetic EMRs of infected patients who were exposed to a fictitious bioterrorism event, the release of airborne tularemia in the restrooms at a sporting event [5]. The synthetic victims (i.e., infected patients) were 4 to 89 years old and included both males and females. The victims' demographics were chosen from the typical demographics of individuals using the luxury boxes at a baseball game. We created synthetic EMRs that reflected the entire timeline of pneumonic tularemia from prodrome, when present, to severe illness, and that were based upon synthesized models of care using information from the literature on the progression of the disease, as well as similar cases of flulike illness and progression to pneumonia present in the set of original background EMRs. Unfortunately, the utility of these synthetic EMRs was limited because, although the injected victims were completely synthetic, the background records were not. Even though these background EMRs were de-identified and date-scrambled via the RBNR algorithm [6], they were considered real enough that they could not be posted on the public health grid [7,8]. In contrast, the completely synthetic EMRs were allowed to be posted. However, the usefulness of the total set of EMRs as a test data set for outbreak detection algorithms was limited to the institutions that had legal and proprietary access to the background data. For this reason, the synthesis of background records became a major part of the EMERGE project. At the time of the creation of this earlier synthetic tularemia outbreak, it was not at all clear that the complexity of the records for a background population would lend itself to synthesis.

We tested, analyzed, and refined methods to extract meaningful information from real EMRs to produce synthetic EMRs, as well as ways to represent the information in a mathematically consistent fashion. After this analysis, we devised a three-part method that would allow automation of the synthesis process with as much fidelity as possible in information content but without compromising details that defined any original patient's identity. This three-part-method consists of: 1) the synthesis of patient identities; 2) the identification of models-of-care in real EMRs; and 3) the adaptation of models-of-care to the synthetic patients. We will describe the processes in detail both for the set of injected tularemia patients and the set of synthetic background patients. Because the pilot age group for the background records was 4-11 yr olds, we will suggest adaptations to this method that we believe are necessary for the creation of data sets beyond this single age group.

### I.b Related Work

There has been considerable research utilizing the information contained in EMRs. Some recent results occur in bio-surveillance [9-13], screening for reportable disease [14-16] and pharmacovigilance [17,18]. However, there has not been a corresponding increase in the availability of non-identifiable complete EMRs for the development or test of algorithms that operate on them.

There have been various efforts at de-identification of medical record information, including the Realistic But Not Real (RBNR) project [6], and recent work on de-identification of clinical notes [19]. Algorithms to de-identify visit records are an active area of research (see, e.g. [20] and references therein) but these algorithms in general do not operate on the entire EMR. As mentioned before, there is some danger in the widespread dissemination of even anonymized or de-identified data. The main risk is that an individual patient or a particular facility or practitioner could potentially be uniquely identified from the conjunction of different types of information within the EMR, such as age group, gender, ethnic group, race, time of medical encounter, or a rare or unusual-for-age diagnosis. Because the medical information itself is not altered by anonymization and date-shuffling algorithms, it is possible that some information in the EMR could be used to identify a patient, a practitioner, or a facility. Proprietary as well as privacy concerns, then, motivate the need to produce synthetic patients that display enough variation in demographic and sanitized medical record information to make identification of the patient and the facility ambiguous.

As a recent effort to model the progression of chronic disease in an individual [21], the Archimedes project models the entire clinical timeline of a fictitious patient, including test and radiology results. Its innovation is in realistically and verifiably modeling the progression of a particular chronic disease, the disease manifestation in test and radiology results, and the outcome of clinical interventions in the individual. It does not model the incidence of the chronic diseases in the population and does not, at this time, model the clinical timelines of patients with infectious disease or injury.

There are many different models for the spread of infectious disease through a population. From a simple lognormal curve [22-24] through the injection of artificial disease outbreaks in simulated time series [25] to the recent population model for the Models of Infectious Disease Agent Study (MIDAS, [26]), they can vary in complexity, scope and purpose. The focus in Project Mimic [25] is in the generation of realistic but totally synthetic time series of case counts. The MIDAS project [26] models the incidence and spread of disease in a completely synthetic population in a geographic area. While it does generate time series of healthcare encounters with verifiably accurate timelines for disease spread, it does not produce EMRs for the synthetic population.

The injection of artificial disease outbreaks into real time-series data, so-called hybrid data, is commonly used to test the effectiveness of disease outbreak detection or clustering algorithms. Typically a series of outbreaks or events is calculated according to an epidemic model, and case counts are added to the real data to simulate the additional cases that would occur as a result of the outbreak. Algorithms can then be tested with and without this outbreak data to gauge their sensitivity (for a general reference see, e.g. [27]). However, injected data is generally limited to time series, or in some cases, injected chief complaint or syndromic data (see, e.g., [28] and references therein) and does not include the complete EMR.

The addition of EMR to the available data sources has fueled recent advances in surveillance methods [29-31]. The ability to engineer outbreaks into synthetic data would allow the development of other surveillance algorithms that can improve sensitivity and specificity of outbreak detections.

The rest of this paper is organized as follows: Section II presents an overview of the method developed, a description of the data set used, and details of the major steps of the methodology: Synthetic Patient Identities and basic information generation, Identification of Closest Patient Care Models and Descriptors, and Adaptation of Patient Care Models. Section III presents the results, and we finish with discussion and conclusions in Section IV.

## II. Methods

### II.a Overview

**Figure 1:**
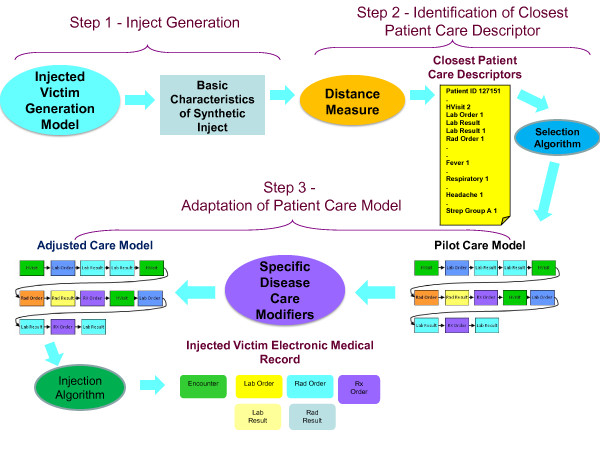
**Injected Victim Generation Technique**.

**Figure 2:**
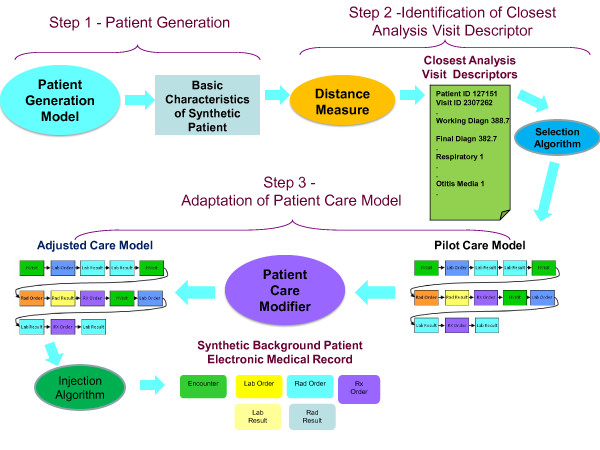
**Background Patient Generation Technique**.

We have developed two methods, one for generating synthetic EMRs for patients included in an infectious disease outbreak (Figure 1) and the second for generating synthetic EMRs for background patients (Figure 2). While these two methods share many features, there are significant differences between them. When describing the methods below, we will point both to similarities and differences between the two techniques.

The synthesis of EMRs for either an injected disease outbreak or for a set of background medical records begins with the determination of who becomes ill (or injured), when they become ill (or injured), and what diseases (or injuries) are the underlying causes of their seeking medical care. In this paper, we will refer to these simulated patients with the disease of interest as the victims. Therefore, the two techniques may be called respectively the *Injected Victim Generation Technique *(Figure 1), and the *Background Patient Generation Technique *(Figure 2).

Once the basic information about patients has been synthesized (date of birth, gender, etc.), the next step is the identification of the care patterns that the synthetic patients would receive. This care pattern is defined as the sequence of health-care events that the patient experiences and it is used to create entries in the synthetic EMR. These EMR entries may include laboratory test orders/results, radiology orders/results, and prescription orders, as well as the clinical history such as working and final diagnoses.

After an appropriate care pattern is identified from the care patterns present in the real EMR data set - the method is described in detail in [49], the creation of the full EMR for the synthetic patient can proceed. The EMR creation steps for the *Injected Victim Generation *differ somewhat from those for the *Background Patient Generation*. Both include the time-stamping of various parts of the EMRs so that the visit time of the synthetic patient and the timeline of the care pattern match, although with some added noise. Both include the assignment of fictitious lab test and radiology order numbers, and the subsequent renumbering of matching results occurs in both techniques. The synthetic background records require sanitization of clinician names, dates and additional reporting events from any records and removal of details, e.g. allusions to specific injury scenarios on radiology results, so that the records could be meaningfully adapted to the synthetic patients. Compared with the background patients, the creation of the synthetic injected disease victim EMRs includes the additional step of altering the information in test and radiology results to simulate those data that would be expected for patients with the particular disease of interest. This step utilizes information such as disease incubation period ranges, frequencies of particular presenting symptoms and signs, disease progression and severity, test results, etc., that may be found in case reports and literature describing outbreaks of the injected disease.

### II.b Definitions

#### Inject

The simulated disease outbreak is called an *inject *because it is injected over the background population. The synthetic EMRs of patients who are infected with the disease of interest will be called *injects*.

#### Victims

The synthetic patients who are infected with the outbreak disease of interest will be called *victims*.

#### Background records or patients

The EMRs of patients other than those with the disease of interest will be called *background records*.

#### Care Model (also called Care Pattern)

A Care Model is defined as the sequence of health-care events that a patient experiences. These care models are identified from real EMR data and described in detail in section II.e.

#### Rx

The abbreviation *Rx *will be used for prescriptions.

### II.c Data Set

We developed the synthetic EMR creation techniques using a dataset that contained 14 months of EMRs from the BioSense [32] program. These data represented 458,346 patients that belonged to five age groups (0-3, 4-11, 12-19, 20-49, 50+ yrs). The records were first anonymized by removing the patients' identifying information (e.g., name, social security number, address). To further protect patient privacy, the records were processed via the RBNR [6] algorithm to shuffle visit dates within a two-week period and patient ages within an age group. The original EMR data were from a metropolitan area in the Midwestern United States.

These BioSense data included seven tables defined as follows: 1) the Analysis Visit Table, which includes patient and visit identifier numbers, age and gender information, a summary of clinical activity, and syndrome and sub-syndrome; 2) the Clinical Activity Table, which includes patient identifiers and detailed records of chief complaint/reason for visit, working diagnoses and final diagnoses; 3) Laboratory Orders; 4) Laboratory Results; 5) Radiology Orders; 6) Radiology Results; and 7) Rx (defined as prescription) Orders. All tables for a particular patient were linked via a patient identification number and a visit identification number. The tables were in the format used by the SAS system [33].

Different subsets of the BioSense data set were used for *Injected Victim Generation *and for the *Background Patient Generation*. For the victims, we extracted from the full data set records of patients who had symptoms similar to those exhibited by patients with any stage of tularemia. Typically, these patients have fever and some combination of cough, chest pain, sore throat, painful respiration, malaise/fatigue, sometimes diarrhea or nausea and vomiting; in later stages they could have hemoptysis, respiratory failure and enlarged lymph nodes. A query on the whole data set to retrieve patients who have combinations of the symptoms described above identified a subset of over 10,000 patients. The rest of the operations for synthetic EMRs of tularemia victims were performed on that subset.

For the *Background Patient Generation*, we extracted from the whole data set the patients from each of age groups: 0-3, 4-11, 12-19, 20-49, 50+ yrs. In further research we concentrated on the 4-11 age group that had 12,599 patients. All data extraction was done via SAS procedures, with most extracted frequencies and tables of data values exported to .csv (comma-separated value) or .xls (Excel spreadsheet) files. We constructed the progression of illness and created patient identities for the injected tularemia patients, constructed the synthetic patient timelines and identities for the background data, and wrote the synthetic EMRs for both sets of patients using Matlab functions. Most of the data analyses and comparisons were performed using Matlab [34]. The computing time necessary for these steps was minimal, on the order of a few minutes.

### II.d Step One: Synthetic Patient Identity and Basic Information Generation

The first major step of our method (see Figure 1 and Figure 2) is the generation of the synthetic patient identities and basic information about the synthetic patients. Because there are some differences in the method for *Injected Victim Generation *and the *Background Patient*, these will be described separately in the next two sections.

#### Injected Victim Record Generation (Figure 1)

In a simulated disease outbreak scenario, the disease is typically modeled to infect a target population, which may be defined geographically, demographically, and by the scenario itself. The method of infection and type of disease dictates the epidemic curve, the type and severity of symptoms, and the disease progression. For example, in the simulated bioterrorist release of tularemia, the release of airborne tularemia occurred in the restrooms near the luxury boxes at a summer sporting event. The timelines for the initial prodrome and then full-blown illness were taken from values in the literature [35,36] that were identified for primary pneumonic tularemia. The expected symptoms, signs, and progression of disease for the simulated victims were adapted from case studies and disease descriptions in the literature (e.g., [36]). For the synthetic tularemia outbreak, we considered patterns of care and produced synthetic victims for the entire metropolitan area and all age groups present at the sporting event (all groups except for 0-3 year olds).

The timelines and illness progression for the synthetic tularemia victims were dependent on the dose of bacterial particles to which each victim was exposed, age, and other disease information found in the literature [35-40]. Because the simulated exposure was to take place in the restrooms at a sports venue, the bacterial dosages for women were slightly higher than for men based on studies showing that women generally spend longer in the restroom than men [41]. A small subset of the victims sought care for influenza-like prodrome symptoms from 4 to 7 days after exposure and returned with increased severity of symptoms 14 to 18 days later. Most victims sought care 18 to 22 days after exposure. We synthesized these patients with demographic information and chief complaints. Using information from literature describing the disease presentations and progression [35-37,40], we compiled a list of symptoms, signs, and their probability of occurrence in patients infected with the disease. For the 10% of the patients who sought care for the initial flu-like illness, symptoms were fever/chills (95% of patients), cough (non-productive) (38%), headache (45%), sore throat(15%), malaise/fatigue (50%), muscle aches(25%), chest pain (20%), nausea and vomiting (20%), joint pain (15%), abdominal pain (10%), and painful pink eye (5%). For severe illness, occurring 18-22 days after exposure, the possible symptoms were fever/chills (in 85% of patients), difficulty breathing (in 30% of patients), shortness of breath (in 24% of patients), hemoptysis (in 11% of patients), respiratory failure (in 12% of patients), rash (30%), chest pain (40%) and cough (65%). Of the people seeking care for severe illness, 10% were chosen to have fever, abdominal pain, nausea, vomiting, and diarrhea, as these are symptoms of sepsis that are particular to tularemia [35].

Using the syndromes and sub-syndromes assigned to the injected patients and a distribution of additional patient attributes that we compiled from the literature, we produced a victim descriptor for each patient. The victim descriptor included the basic characteristics of the injected patient: age, gender, race, ethnic group, certain syndromes and sub-syndromes related to tularemia (described in the previous paragraph). These victim descriptors were then passed to the next step (identification of closest care models) of the EMR synthesis procedure.

It is worth noting that the real EMRs we used did not contain any diagnoses of tularemia. Thus, using expert medical opinion, we searched for patterns of care that matched the sequence of symptoms that were generated in the course of simulating the illness progression in each synthetic victim. This procedure is described in detail in section II.e. In some cases patterns of care could be found that were quite close to what was expected for tularemia and in some cases there were discrepancies; we describe the required adjustments to the data in section II.f.

#### Synthetic Background Records Generation (Figure 2)

There is no single scenario or target population when the intention is to produce synthetic background records. The underlying causes of symptoms and reasons for seeking care are not known and need to be imposed on the synthetic patients in an approximation of those that appear in the real EMRs. However, the real EMR is an inexact reflection of the underlying condition of a patient. Even the most carefully entered EMR has been filtered by medical personnel subject to insurance rules and hospital or office protocols (see, e.g. [42]). To synthesize the background patients, these real EMRs must be analyzed for the probable underlying illnesses and injuries and their timelines. The pilot group of patients for the synthetic background records was the 4-11 year age group. We further restricted the patterns of care and synthetic patient demographics to a 5-county area including the metropolitan area and some surrounding suburban areas. We used the timelines in the clinical activity record as the data to drive creation of the synthetic patient identities.

In order to implement our data-driven approach to create synthetic EMRs from real EMRs, we needed to select a driving data element (i.e., an independent variable).that could be used to determine the other linked patient information for the background patients. For this driving data element, we considered using syndrome classification, sub-syndrome classification, 3-digit ICD-9 final diagnosis code [43], and chief complaint/reason for visit. The percentage of each data element present in the real EMRs is listed in Table 1. The percentage of patients who had each data element present was important to the choice of driving variable. If a particular data element is missing in a majority of the patients but is used as a driving variable, fewer patients comprise the pool of records that can be mined for information.

**Table 1:** Data elements present in the real data set.

Data Element	% of visits in model data
Chief Complaint or Reason for Visit	85.16
Sub-syndrome	83.52
Syndrome	54.41
Final diagnosis ICD-9 code	99.72

**Table 2:** Possible ICD-9 codes associated with the chief complaint "sore throat."

ICD-9 Code	Description
034.0	034.0 STREP SORE THROAT
079.99	079.99 VIRAL INFECTION NOS
382.9	382.9 OTITIS MEDIA NOS
462	462 ACUTE PHARYNGITIS
463	463 ACUTE TONSILLITIS
465.9	465.9 ACUTE URI NOS
466.0	466.0 ACUTE BRONCHITIS
473.9	473.9 CHRONIC SINUSITIS NOS
486	486 PNEUMONIA, ORGANISM NOS
528.0	528.0 STOMATITIS
786.07	786.07 WHEEZING
	

This does not include misspellings or abbreviations.

Another, even more relevant, consideration is the definition of the mapping from the driving data element to illness. By the mapping from data element to illness, we mean the identification of a single value of a data element with a particular illness, injury or condition. Ideally, the data element used to define the patients would have a clear and easily recognized relationship to underlying illness or injury. The mapping from data element to illness is "well defined" in the real data if the patient visits are grouped by a single value of this data element and if it differs very little in the underlying illness or condition that can be inferred from the information in the medical record. This is akin but not equal to the notion of specificity; a well-defined mapping minimizes the inclusion of dissimilar records associated to the data element. This mapping also has to be well-defined in the inverse sense, meaning that there is little *overlap *in the underlying illness or injury (as manifested in the EMR) for patient visits that are grouped by *different *values of the data element. This notion is akin but not equal to sensitivity; a well-defined inverse mapping minimizes the number of data elements that are associated with similar electronic medical records. If such a mapping is reasonably well-defined in both directions, the timelines of different values for this data element could be mimicked to impose the entire spectrum of illness and injury to a synthetic population via an automated process. If the mapping is not well-defined, using this data element to drive the synthetic population creates several problems. We illustrate the possible problems by considering the mapping for various candidate data elements in the EMR.

First let us consider the use of chief complaint, syndrome or sub-syndrome as the driving data element. Because of the variations in the spelling and abbreviations in chief complaints, the additional step of natural language processing (e.g. [44,53]) is necessary to categorize chief complaints in advance of associating them with particular illnesses. Even with the use of a natural language processor, this association is not particularly well-defined. For example, the chief complaint *sore throat *(and many variations) appears quite often in this age group. However, sore throat can define or be present in multiple illnesses. Table 1 shows the diagnoses that are extracted from the real data for the chief complaint *sore throat *for this age group. Similarly, the 10 syndrome definitions and 68 sub-syndrome definitions do not exactly define an illness or injury. Although multiple chief complaints, syndrome definitions and sub-syndrome definitions can more accurately describe the illness or injury, this mapping is still inexact.

There are different possible illnesses or injuries that may be associated with a chief complaint, syndrome or sub-syndrome and the inverse is also true: there are many possible chief complaints for each underlying illness (or injury). To illustrate this, we extracted all patient visits with a single final diagnosis code of strep throat (ICD-9 code 034.0) from both emergency and outpatient cases in the subset of 4-11 year old patients from the real data set. Among emergency cases, there were 49 different chief complaint strings, taken from a list of 28 different single complaints (e.g. fever, nausea, neck pain, cold, cough, stuffy nose, abdominal pain, vomiting, sore throat or variations such as throat pain, throat sore, etc. as well as others). Although sore throat (and variations) appeared quite often in the strings, there were 26 complaints (53%) that did not contain sore throat, throat pain, or other such variations anywhere in the complaint text. In the outpatient cases, there were 62 different reasons-for-visit, with 30 different single complaints. Of these, 9 (14.5%) did not contain sore throat, throat pain or other such variations anywhere in the reason-for-visit text. This ill-defined mapping translates to the following problem with using chief complaint as the driving data element. If patients were synthesized only from timelines of particular chief complaints, the variety of underlying causes would have to be synthesized independent from, but consistent with, the chief complaints. This would require either considerable expert input on the possible conditions associated with those chief complaints or the categorization of ICD-9 codes and/or syndromes and sub-syndromes by chief complaint.

**Figure 3:**
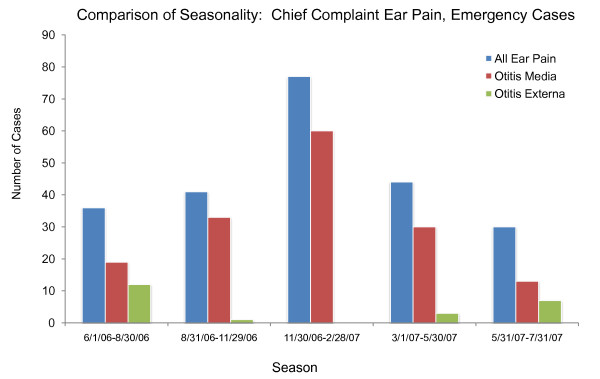
**Seasonal Differences between two underlying causes of the chief complaint "ear pain" (or "ear ache") from the real data, along with seasonal distribution of "ear pain." Note that using the timeline for "ear pain" to drive patient visit data would not recover the seasonal variation of either cause**.

**Figure 4:**
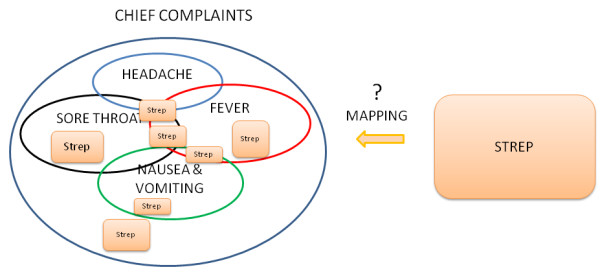
**The inverse map from underlying illness to Chief Complaint is not well defined; multiple overlapping and non overlapping chief complaints contain the strep throat cases**.

**Figure 5:**
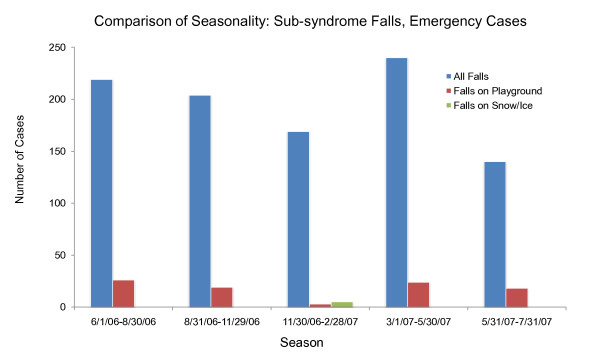
**Seasonal distribution of two underlying causes of the subsyndrome "falls." Using subsyndrome alone to generate synthetic patients would not recover the seasonal variation of different types of falls**.

An additional difficulty with using chief complaints, syndromes or sub-syndromes is that temporal variations in specific conditions would be inherited from the general category of data elements and may not match those for specific illnesses in the real data. For example, *ear pain *may be associated with *Otitis externa *or *Otitis media*. Otitis externa exhibits different seasonal incidence from otitis media (see Figure 3) but, if chief complaint were used to drive the synthetic patient data, neither seasonal variation would be apparent. The inverse mapping from illness to chief complaint is also important for this reason. If cases for a particular illness (e.g. strep throat - see Figure 4) are spread across more than one chief complaint (as they usually are), it is difficult to recover the timeline for this illness reliably from the chief complaints. Conversely, different timelines for variations in a syndrome or subsyndrome (e.g. falls) cannot be recovered by using the timeline for the syndrome or sub-syndrome alone (see Figure 5).

Next, let us consider using ICD-9 code as the driving data element. Although the final diagnosis ICD-9 code does not always completely describe or define the underlying illness or injury of a patient [45,46], there is a relatively clear relationship between final diagnosis ICD-9 code and the care that a patient would receive, as well as possible chief complaints, syndromes and sub-syndromes. There is some overlap in the mapping from underlying illness or injury to ICD-9 code, because of inexact coding, coding conventions and insurance reimbursement rules. The timelines for specific ICD-9 codes should reflect, to a certain degree, seasonal or sporadic variations in disease and injury occurrences. We also note that this is the data element that is least likely to be omitted in an EMR. Thus, we settled on the final diagnosis ICD-9 code as the "driving" data element. In other words, we replicated statistical and mathematical properties of the ICD-9 code time series and used those to impose those illnesses and injuries on the synthetic patients. All attributes of the patients were chosen by using the final diagnosis ICD-9 code as the determining variable for age (within the age group), gender, race/ethnic group, and syndrome and sub-syndrome classifications. The use of a single ICD-9 code as the driving data element proved to be sufficient for this age group because the majority of the visits in the real data exhibited a single ICD-9 code. Other age groups may require a multi-dimensional ICD-9 code map, depending on the characteristics present in the real data.

The BioSense dataset contained 6426 clinical activity records for Emergency (ER) patients and 3248 records for outpatient (OP) patients. Of the 6426 ER visit records, 65% of the visits had 29 different final diagnosis ICD-9 codes with 100 or more patient visits. These 29 included ICD-9 codes 873., 382., 493., 465., and 034 as the codes with the most patient visits; those 5 ICD-9 codes comprised 28% of the patient visit records. However, we reconstructed timelines for 295 ICD-9 final diagnosis codes for the synthetic ER patients. Of the 3248 OP visits, 43% of the visits had 14 different final diagnosis ICD-9 codes with 60 or more patient visits. These 14 included codes 382, 079, 462, and 034.

Because the primary diagnosis and any secondary diagnoses were not differentiated in the BioSense dataset we received, we used a convention to define the primary diagnosis. For each patient in the real data set, we sorted the final diagnosis codes in numeric order, with the exception that specific injury codes (prefixed E) were excluded from occurring as primary diagnoses. The first code (after alphanumerically sorting) was defined to be the primary diagnosis. The time series of healthcare events related to this primary diagnosis were mimicked to produce the synthetic patient timelines. Furthermore, we verified that this set of primary diagnosis ICD-9 codes did not contain diagnoses that were either considered rare by a subject matter expert or never appeared as a single primary diagnosis. This procedure thereby assured that sorting the ICD-9 codes and using the first code as the primary diagnosis did not omit any other potential primary diagnoses.

Rare diagnoses, diagnoses that are unusual in this age group, and diagnoses that included multiple congenital conditions were excluded from consideration because an individual could be identified from such a diagnosis together with knowledge of the particular region. Producing multiple records from these ICD-9 codes in order to remove the possibility of identification would yield a higher-than-usual incidence of rare or unusual conditions in the synthetic data. Although it could be argued that exclusion of rare diagnoses reduces the fidelity and realism of the synthetic data, a data set without the *particular *rare diagnoses that were excluded is not unrealistic. Care models for randomly chosen rare or unusual illnesses or conditions could be synthesized using expert opinion to improve the realism of a large synthetic data set.

We extracted the timelines of truncated (without detail codes after the decimal point) final diagnosis ICD-9 codes from the clinical activity records of the real data and reconstructed similar (but not identical) timelines in two ways, dependent on the sparseness or richness of the data stream. For time series with on average more than 1 case per week, we performed a Haar wavelet-2 [47] reconstruction to de-noise but replicate the frequencies in the time series. We tested reconstructions using both Haar and Daubechies wavelets as well as a reconstruction based on nonlinear prediction (see, e.g. [48] section 4.2). The Haar wavelet-2 reconstruction yielded the best prediction in terms of root-mean-square (rms) values and total numbers of cases. For time series with less than 1 case per week, we calculated seasonally-varying weekly Poisson parameters. We reconstructed those time series by taking Poisson draws from the specified distribution, and by using day-of-week probabilities from the actual time series (again, adjusted seasonally) to assign a day of the week to the cases. Demographic data was extracted from the real data, keyed from primary final diagnosis ICD-9 code, and assigned to each synthetic patient based on the primary ICD-9 code. The birth date for each synthetic patient was chosen randomly based on the synthetic patient's age to further distance each synthetic patient's identity from that of a real patient. Additional ICD-9 codes were assigned to the synthetic patients according to a seasonally varying multivariate distribution dependent on primary diagnosis ICD-9 code. Sub-syndromes and syndromes were assigned to the synthetic patients dependent on all the final diagnosis ICD-9 codes using the patterns extracted from the real EMR data. The patient's visit descriptor with the basic characteristics of the synthetic patient's visit includes patient's age, gender, race, ethnic group, syndromes, sub-syndromes, and final diagnosis ICD-9 codes. At this point, the basic visit descriptors for the synthetic patients contained enough information for the identification of closest patient care descriptors (Figure 2) to proceed. We note that the chief complaint is that of the pattern of care rather than chosen from a distribution based on the final diagnosis ICD-9 code. Since the synthetic background patients were created from ICD-9 code streams of the real patient population, the patterns of care that this population would receive were present in the real data. We note that the synthetic patients mimicked rather than exactly duplicated the real patient population: no synthetic patient matched a real patient exactly in age, gender, demographic variables and visit information (ICD-9 codes, syndromes and sub-syndromes) although as a group the age, gender, demographic variables and ICD-9 diagnoses displayed the same statistical distributions as the real data. Although this was precisely the intent of producing completely synthetic patient identities, this inexact matching made locating appropriate patterns of care for the synthetic patients complicated. This method cannot match the multidimensional data fidelity of, for example, a shuffling and anonymization algorithm (e.g. RBNR). However, it does reproduce many of the multiple dimensions present in the real data (see verification results here and in [49]) and mitigates many objections to dissemination of anonymized and shuffled data. In the next section we describe, in detail, the methods for identifying patterns of care for both the injected disease victims and for the background synthetic patients. The identification of the pattern of care for the background patients is another safeguard for anonymization. Although it was not necessary for this particular data set, the algorithm that chooses the care model can be adapted to exclude any exact matches in demographics, ICD-9 codes, syndromes and sub-syndromes from consideration.

### II.e Step Two: Identification of Closest Patient Care Models and Descriptors

Step Two performs the identification of the medical care that each of the synthetic patients would receive. Basic characteristics of the synthetic patient are coming from the *Injected Victim Generation Model *(see Figure 1)/*Background Patient Generation Model *(see Figure 2). They include syndromes, sub-syndromes, age, gender, race and ethnic group of each synthetic patient. For the background patient EMRs, these characteristics additionally include visit information and the final diagnosis ICD-9 codes.

Step 2 for *Injected Victim Generation *operates on Patient Care Descriptors (Figure 1) and for *Background Patient Generation *it operates on *Analysis Visit Descriptors *(Figure 2). A distance measure is defined, and the closest *Patient Care Descriptor*/*Analysis Visit Descriptor *is identified in the original data set. The information from this *Patient Care Descriptor*/*Analysis Visit Descriptor *is next given to major step 3: *Adaptation of Patient Care Model*. The steps mentioned need to be executed separately for each synthetic patient (and for each visit in case of *Background Patient Generation*. These steps are described in detail in Section II.f.

Before the above steps can be executed, Patient Care Models, Patient Care Descriptors, and Analysis Visit Descriptors need to be extracted from the EMR data set. This is a process that is performed only once for the whole data set. It is described in detail later in this section. The computation of Patient Care Models and Patient Care descriptors is computationally expensive: it takes about 30 sec per patient. For 10,000 patients such a computation takes about 80 hours on a 32-bit PC.

#### Patient Care Models

A *Patient Care Model *[50] is a sequence of all the health care encounters (that we will also call events) that were present in the original EMR data set for a given patient. The care model consists of up to seven types of events: 1) Analysis visit (abbreviated AVisit); 2) Laboratory order; 3) Laboratory result; 4) Radiology order; 5) Radiology result, 6) Prescription order (abbreviated Rx), 7) Death event - if applicable. The events in the extracted care model are sequentially ordered based on the dates and times they occurred (as present in the data).

**Figure 6:**
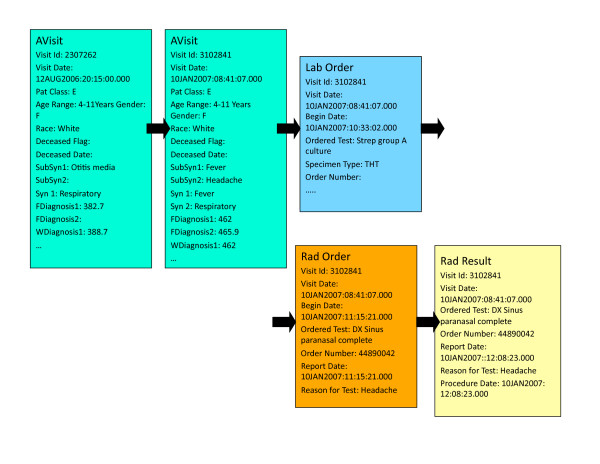
**Example of Patient Care Model**.

The AVisit contains the information about a given patient visit including patient's demographic data, visit identification number, visit date, syndromes, and sub-syndromes. For *Background Patient Generation*, AVisit also incorporates working diagnoses ICD-9 codes and final diagnoses ICD-9 codes. The demographic data consists of patient birth date, age, race, ethnic group, and gender. Inside the laboratory orders, there is information on each of the laboratory tests that were ordered during a given visit, including date and time. In the laboratory results, there is information on each of the labs with, for example, bacteria identified or information that the test was negative. Individual Patient Care Models are of different lengths (depending on the number of visits and specific information in laboratory orders, laboratory results, radiology orders, radiology results and Rx orders). People who came only once and did not have any laboratory or radiology orders have patient care models with one record. People who came many times and had many laboratory or radiology orders and results have very lengthy care models (hundreds of records). Figure 6 shows the Patient Care Model for a 4-11 year old girl with two AVisits, one laboratory test (for Strep), one radiology test (DX Sinus paranasal complete) and one radiology result (DX Sinus paranasal complete).

#### Patient Care Descriptors

*Patient Care Descriptors *summarize Patient Care Models. For each Patient Care Model, one Patient Care Descriptor is computed. For *Background Patient Generation*, Patient Care Descriptors consist of individual *Analysis Visit Descriptors *(see Table 3 and Table 4) that describe a given visit in detail, including any laboratory and radiology tests related to that visit. The Analysis Visit Descriptor for visit 2307262 is shown in Table 3. It has many empty fields because there were no laboratory or radiology orders or results related to that visit. The Analysis Visit Descriptor for visit 3102841 (Table 4) is more complicated because it contains information on one laboratory order, one radiology order and one radiology result. If a given patient (e.g., the one from Figure 3) had two visits, his or her Patient Care Descriptor will consist of two Analysis Visit Descriptors chronologically ordered. For that patient, the Patient Care Descriptor encompasses the Analysis Visit Descriptor for visit 2307262 and the Analysis Visit Descriptor for visit 3102841. Should the patient visit the hospital often and have 30 analysis visits, his or her Patient Care Descriptor would consist of 30 Analysis Visit Descriptors.

**Table 3:** Example Analysis Visit Descriptor (for Visit 2307262).

**Patient Id**	127151
**Visit Id**	2307262
**Visit Date**	12AUG2006:20:15:00.000
**Patient Class**	E
**Age Range**	4-11 Years
**Gender**	F
**Race**	White
**Ethnic Group**	Not Hispanic or Latino
**Deceased Flag**	
**Deceased Date**	
**Syndrome 1**	Respiratory
**Syndrome 2**	
**...**	...
**Subsyndrome 1**	Otitis media
**Subsyndrome 2**	
**...**	...
**Working Diagnosis 1**	388.7
**Working Diagnosis 2**	
**...**	
**Final Diagnosis 1**	382.7
**Final Diagnosis 2**	
**...**	
**Number Laboratory Orders**	
**Number Laboratory Results**	
**Number Radiology Orders**	
**Number Radiology Results**	
**Number Rx Orders**	
**Botulism Like**	
**Fever**	
**...**	
**Respiratory**	1
**...**	
**Abdominal pain**	
**...**	
**Otitis media**	1
**...**	
**Blood Culture**	
**MRSA Culture**	
**...**	
**Strep Group A Culture**	
**Urine Culture**	
**DX Chest**	
**DX Sinus Paranasal**	

**Table 4:** Example Analysis Visit Descriptor (for Visit 3102841).

**Patient Id**	127151
**Visit Id**	3102841
**Visit Date**	10JAN2007:08:41:07.000
**Patient Class**	E
**Age Range**	4-11 Years
**Gender**	F
**Race**	White
**Ethnic Group**	Not Hispanic or Latino
**Deceased Flag**	
**Deceased Date**	
**Syndrome 1**	Fever
**Syndrome 2**	Respiratory
**...**	...
**Subsyndrome 1**	Fever
**Subsyndrome 2**	Headache
**...**	...
**Working Diagnosis 1**	462
**Working Diagnosis 2**	
**...**	
**Final Diagnosis 1**	462
**Final Diagnosis 2**	465.9
**...**	
**Number Laboratory Orders**	1
**Number Laboratory Results**	
**Number Radiology Orders**	1
**Number Radiology Results**	1
**Number Rx Orders**	
**Botulism Like**	
**Fever**	1
**...**	
**Respiratory**	1
**...**	
**Abdominal pain**	
**Abdominal cramps**	
**...**	
**Headache**	1
**...**	
**Blood Culture**	
**MRSA Culture**	
**...**	
**Strep Group A Culture**	1
**Urine Culture**	
**DX Chest**	
**DX Sinus Paranasal**	2

For *Injected Victim Generation *because lower level of detail was available, Patient Care Descriptors summarize all the care given to a patient without distinguishing for which Avisit the care occurred. An example Patient Care Descriptor for *Background Patient Generation *is shown in Table 5. In addition to patient identification number, ethnic and demographic information, it contains the numbers of AVisits, laboratory orders and results, radiology orders and results, Rx orders, all the syndromes, sub-syndromes, types of laboratory and radiology tests, and test results.

**Table 5:** Patient Care Descriptor for Patient 127151.

**Patient Id**	127151
**Age Range**	4-11 Years
**Gender**	F
**Race**	White
**Ethnic Group**	Not Hispanic or Latino
**Deceased Flag**	
**Number AVisits**	2
**Number Laboratory Orders**	1
**Number Laboratory Results**	
**Number Radiology Orders**	1
**Number Radiology Results**	1
**Number Rx Orders**	
**Botulism Like**	
**Fever**	1
**GI**	
**Hemorrhagic Illness**	
**Localized Cutaneous Lesion**	
**Neurological**	
**Rash**	
**Respiratory**	1
**Severe Illness or Death**	
**Specific Infection**	
**Other**	
**Abdominal pain**	
**Abdominal cramps**	
**...**	
**Headache**	1
**...**	
**Otitis media**	1
**...**	
**Blood Culture**	
**MRSA Culture**	
**...**	
**Strep Group A Culture**	1
**Urine Culture**	
**DX Ankle**	
**DX Chest**	
**...**	
**DX Sinus Paranasal**	2
**...**	

#### Extraction of Existing Patient Care Models and Descriptors

**Figure 7:**
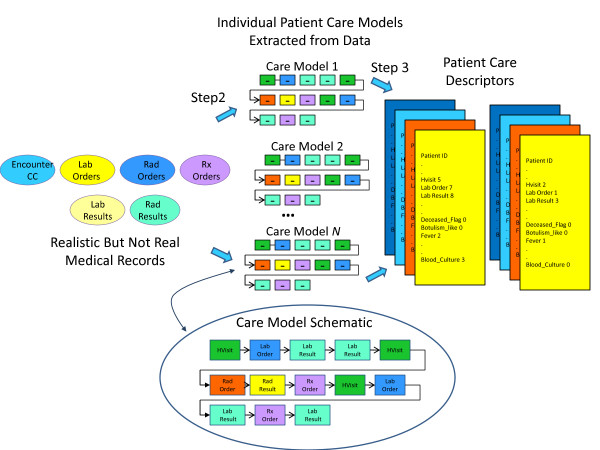
**Identification of Existing Patient Care Models and Patient Care Descriptors for Injected Victim Generation**.

**Figure 8:**
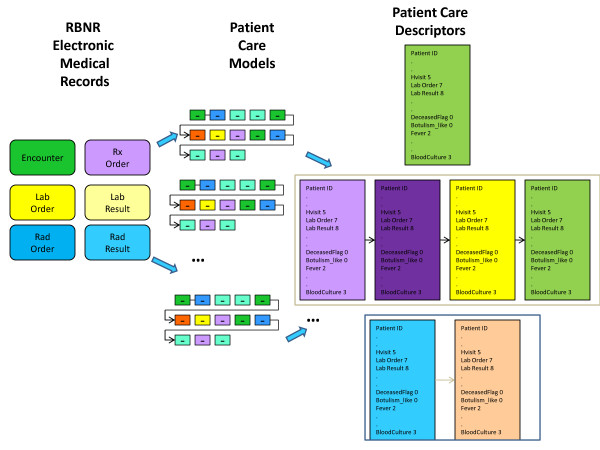
**Identification of Existing Patient Care Models and Patient Care Descriptors for Background Patient Generation**.

The goal of the methodology developed is to derive, from the available real EMR data, a care model of how the patients are treated. This method has the following main steps (Figures 7 and 8):

1) Build *Patient Care Models *- sequences of patient care events for each patient.

2) Build *Patient Care Descriptors *summarizing Patient Care Models.

Figure 7 shows the procedure for Injected Victim Generation, and Figure 8 shows the procedure for Background Patient Generation. The first part of the procedure is exactly the same in both cases: Patient Care Models are computed from the EMRs anonymized by the RBNR algorithm and; all seven tables (described in Section II.c) with RBNR records are accessed in order to extract all the information pertaining to a given patient.

For Synthetic Inject Records Generation (Figure 7), the Patient Care Descriptors are determined next, one descriptor per patient. An example Patient Care Descriptor was shown in Table 5. However, for Background Patient Generation (Figure 8), the procedure is slightly more complicated: after the calculation of Analysis Visit Descriptors (one descriptor per analysis visit), they are grouped into Patient Care Descriptors. The Analysis Visit Descriptors are ordered by occurrence date in the Patient Care Descriptors. The process of building both Analysis Visit and Patient Care descriptors is completely data driven: if there are *n *different microorganisms identified in the data set, there will be *n *corresponding fields in the descriptor; if there are *m *different types of laboratory tests in the data set, there will be *m *corresponding attributes in the descriptor.

#### Identification *of Closest Descriptors*

The information coming from Step One into Step Two is different for the Injected Victim Generation (Figure 1) and Background Patient Generation (Figure 2). For Injected Victim Generation, this information does not include any data about individual visits for a given patient (Figure 1). For our tularemia example, this information only contains certain syndromes and sub-syndromes related to tularemia-like illness at different stages of disease progression (e.g., fever, cough, headache, sore throat, malaise and fatigue, muscle aches, chest pain, nausea and vomiting, abdominal pain, difficulty breathing, respiratory failure, rash). This is the reason for having the matching algorithm, subsequently described, operate on Patient Care Descriptors.

In the case of *Background Patient Generation*, the information coming from Step One into Step Two is much richer: it contains a high level description of every patient visit including syndromes, sub-syndromes, and final ICD-9 codes (Figure 2). Because of the high granularity of this information, it is possible to operate on individual patient visits, and hence the matching algorithm uses Analysis Visit Descriptors.

For *Injected Victim Generation*, the process of finding the closest set of patient care descriptors is shown as step 2 on Figure 1. A distance measure is computed between the inject descriptors and existing Patient Care Descriptors. The few closest descriptors are identified (*Distance Measure*). Next, the patient care models corresponding to the Closest Patient Care Descriptors are selected from the background data (*Closest Care Model*).

A distance measure is used to identify the closest (minimum distance) Patient Care Descriptor to the desired inject. Gower's General Similarity Coefficient [51] and several Euclidean distances were investigated. The distance measure needs to be well-tuned to the illness of interest because its goal is to identify the patients who have attributes similar to those of the inject. Certain attributes are not related to tularemia, and therefore their presence or absence is inconsequential and for that reason these attributes will not be used in the distance measure. An example of such an attribute is *Cardiac dysrhythmias *because whether or not a person has *Cardiac dysrhythmias *sub-syndrome, this person may have symptoms similar to those of tularemia and the treatment will also be similar.

The Euclidean distance operates on the following 23 attributes: Syndrome attributes - Fever, Gastrointestinal, Rash, Respiratory; and Sub-syndrome attributes - Abdominal pain, Alteration of consciousness, Chest pain, Convulsions, Cough, Diarrhea, Dyspnea, Headache, Hemoptysis, Hemorrhage, Influenza-like illness, Lymphadenopathy, Neoplasms, Malaise and fatigue, Nausea and vomiting, Respiratory failure, Septicemia and bacteremia, Upper respiratory infections, Severe illness or death.

The Euclidean distance, *dist(I, P)*, between the inject descriptor I, and existing Patient Care Descriptor P is defined as:

(1) $$dist\left(I,P\right)=\sqrt{\Sigma_{i=1}^{23}w_{i}{\left(I_{i}-P_{i}\right)}^{2}}$$

Where *w*_*i *_= 1 for 1 ≤ = *i *≤ = 22, and *w*_23 _= 0.1; I_*i *_and P_*i *_signify the value of a given attribute (from the attributes specified in the previous paragraph) for the inject descriptor and patient care descriptor, respectively. The attribute for which the weight of 0.1 is used is *Severe illness or death*. One might ask why one of the attributes used in the distance measure is neoplasms: neoplasms have nothing to do with tularemia, so why use this sub-syndrome in the distance measure? The fact that they have nothing to do with tularemia is precisely why they are needed. Before we included neoplasms in the distance measure, most of the closest patient care descriptors retrieved for injects with malaise and fatigue had neoplasms. We wanted to exclude patients with neoplasms because their care models are too different from those with tularemia. Therefore, for all the injects, we set the neoplasms attribute to 0, and using the distance measure in Eq. (1), we were able to retrieve patient care descriptors that had no neoplasms as diagnosis. Ten nearest neighbors (in terms of the Euclidean distance), from the same age group as the inject are retrieved. These nearest neighbors are processed by the *Specific Disease Care Modifier *(described in Section II.f).

**Figure 9:**
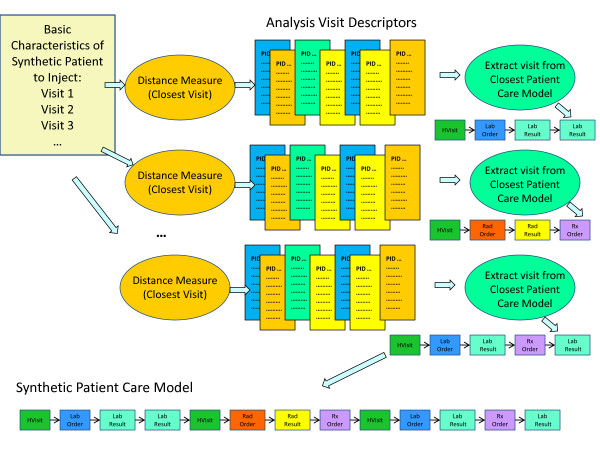
**Identification of closest analysis visit descriptor to a synthetic visit descriptor**.

For the Background Patient Generation, the procedure described below is performed for every synthetic visit of every synthetic patient. The goal is to find, for each synthetic visit, a visit that is as similar as possible in the real data. Figure 9 depicts how the closest analysis visit descriptor to a synthetic visit descriptor is determined. The basic characteristics of the synthetic patient, in the form of information about synthetic visits for a given patient, are produced by *Patient Generation Model *(see Figure 2). Each synthetic visit is characterized by final diagnosis ICD-9 codes, syndromes, sub-syndromes, age, gender, race and ethnic group of the synthetic patient. A distance (the distance measure used is described further in this section) is computed between a given synthetic visit and all the analysis visit descriptors resulting in a set of closest (i.e. minimum distance) analysis visit descriptors. All the information about that visit is extracted from the corresponding Patient Care Model.

We define a distance measure between a synthetic visit and an Analysis Visit Descriptor that is a combination of weighted Euclidean distance and Jaccard distance [52]. The Jaccard index (or similarity coefficient) measures similarity between two sets, and is defined as the size of the intersection divided by the size of the union of the sets:

(2) $$J(A,B)=\frac{\left|A\cap B\right|}{\left|A\cup B\right|}$$

The Jaccard index is a useful measure of similarity in cases of sets with binary attributes because it takes into account not only how many attributes agree but also how many disagree. If two sets, A and B, have *n *attributes each, then Jaccard index measures the attribute overlap that A and B share. The Jaccard distance is then defined as:

(3) $$J_{dist}(A,B)=1-J(A,B)=\frac{\left|A\cup B\right|-\left|A\cap B\right|}{\left|A\cup B\right|}$$

We defined and used the following distance measure to identify the closest Analysis Visit Descriptor to a given synthetic visit descriptor:

(4) $$dist(A,S)=J_{dist}(A,S)+\frac{E_{dist}(A,S)}{70}$$

where *A *stands for Analysis Visit Descriptor, *S *- for Synthetic Visit Descriptor, and *E*_*dist *_- for Euclidean distance. Jaccard distance is computed on all binary attributes: truncated final diagnosis ICD-9 codes, syndromes, and sub-syndromes; Euclidean distance is only computed on a non-binary attribute (age). J_dist_(A,S) has a value between 0 and 1 (inclusive) and E_dist_(A,S) has a value between 0 and 7 (inclusive). In order to make both distances contribute the same to the final distance measure, E_dist_(A,S) would have to be divided by 7. When designing dist(A,S) we wanted the age attribute to contribute only 1/10 as much as the J_dist_(A,S). Therefore, we need to divide the Euclidean distance by 70 in Equation 4 above. Because it is most important to have agreement on as many final diagnosis ICD-9 codes, syndromes, and sub-syndromes as possible, the agreement in age between Analysis Visit Descriptor and Synthetic Visit Descriptor is secondary.

Next we examine the search methods and rationale behind assignment of a specific care model to a synthetic patient. These processes differed both in automation level and in specific procedures for the synthetic tularemia victims and for the synthetic background patients.

### II.f Step Three: Adaptation of Patient Care Model

#### Care Model Assignment and Adaptation

The third step in the process (see Figure 1 and Figure 2) is adapting the chosen care model to the specific synthetic patient and actually writing the electronic medical records. During the development of this project, the methodology evolved significantly between the creation of the injected tularemia victims and the creation of the synthetic background records. We were able to automate nearly all of the steps for adaptation of care models for the synthetic background patients but there was considerable non-automated effort in the adaptation of the care models for the synthetic tularemia victims.

A medical expert inspected the closest care models that were identified for the synthetic tularemia victims and made recommendations for tests that either needed to be deleted or added, with the assumption that the attending physician might not realize the underlying illness were tularemia. We added either rapid strep tests or influenza tests to some records, but adjusted any results to reflect the absence of either strep or influenza. If respiratory or blood cultures were taken, we modified results to indicate no growth, reflecting the fastidious nature of tularemia in routine cultures. In some cases, we added chest x-rays to the records of patients whose care models did not include them to suggest a physician's possible response to an unexplained severity or persistence of respiratory symptoms. Chest x-ray results were modified to include evidence of pathology that would be common for pneumonic tularemia patients. Because the care models had to be inspected and edited individually and the patients assessed based on the symptoms generated by the injection algorithm, little automation was possible for this phase of the synthetic tularemia injection method. This is the biggest drawback of the methodology proposed.

In the case of Background Patient Generation, the distance measure used to find care models identified up to ten care model candidates for each synthetic background patient. We automated the process of choosing the most appropriate care model from those identified by examining specific ICD-9 codes and exact syndromes and sub-syndromes in the patient visit records and in the care models. The hierarchy was first to try to match all exact ICD-9 final diagnosis codes in the patient visit record. If multiple care models still matched the patient visit record, the algorithm chose by specific sub-syndrome and syndrome codes. If multiple care models still matched after this step, a care model was chosen randomly from the remaining candidates, with equal probability given to each.

The final part of Step Three of the methodology is the adaptation of the entire EMR to the particular patient to assure that there is no exact match between a synthetic and a real patient. This procedure is described in the next section.

#### Injection Algorithm

The injection algorithm unites the patient visit record and the care model to produce a consistent EMR for a synthetic patient so that the EMR has visit-linked entries in the six tables - analysis visit, clinical activity, radiology orders, radiology results, laboratory orders and laboratory results. Because the Rx orders in the real data set were incomplete and sporadic, we did not generate the synthetic Rx orders table. The injection algorithm time-stamps the entries subsequent to the visit date by using the time intervals found in the care model with randomly chosen (uniform) variation. Any radiology or laboratory orders are assigned unique identification numbers, and these numbers are carried through to the radiology and laboratory results if present. The algorithm also produces summaries of clinical activity and sub-syndrome/syndrome information in the format found in the model data and writes these in the analysis visit record. All formats found in the original records are duplicated by the injection algorithm. However, patient zip code, health department identifier, and location are not synthesized for the synthetic background records. The algorithm writes the tables to comma-separated-value (csv) files, preserving leading zeros on ICD-9 codes and producing SAS date formats as well as MS-EXCEL-readable formats.

## III. Results

### III.a Tularemia Injects

The simulated tularemia outbreak resulted in 203 synthetic victims. Of these victims, 19 individuals sought care for prodrome symptoms but none of these synthetic patients were admitted to a hospital at that time. All 203 victims sought care for a severe respiratory illness after 18-22 days; there were 57 synthetic patient admissions and 17 synthetic victim deaths.

As part of the validation process, a medical expert reviewed all the synthetic records and determined that 42 records (i.e. 19% of the inject records) had content problems or inconsistencies. The predominant problem was a string of ICD-9 codes that did not match any of the expected symptoms of tularemia. For example, a patient would be assigned ICD-9 codes of "250.00 DMII WO CMP NT ST UNCNTR", "272.4 HYPERLIPIDEMIA NEC/NOS", "410.71 SUBENDO INFARCT, INITIAL" as well as the ICD-9 code of "486 PNEUMONIA, NOS." Because no cases of tularemia were found in the real data, it was expected that some difficulty would arise with matching the synthetic patient descriptors to an appropriate care model. Of the 221 visit records (for 203 patients), 36 were deemed unsuitable for this reason. Other problems included incompatible ICD-9 codes (2 records), for example, both "786.50 CHEST PAIN NOS" and "786.52 PAINFUL RESPIRATION," exact duplication of real EMR information (2 records), and odd or incorrect ICD-9 codes (2 records) such as a diagnosis of "263.9 PROTEIN-CAL MALNUTR NOS." These 42 records were adjusted manually by editing the fields that were deemed inconsistent, odd, or erroneous. It was noted by the medical expert that the presentation of tularemia in the remaining synthetic records was as expected.

The synthesized electronic medical records included syndrome and sub-syndrome classifications for the injected patients. The severe respiratory illness imposed on the victims was manifested in EMRs that included multiple syndrome and sub-syndrome classifications. Of the 221 visits for 203 victims, over 92% exhibited the fever syndrome, nearly 80% exhibited the respiratory syndrome, and 12% had severe illness or death. There were also over 10% of patients with hemorrhagic illness and over 12% with gastrointestinal syndrome. Sub-syndrome classifications for the injected patients included over 44% with cough, nearly 40% with dyspnea, over 47% with fever, and 19% with pneumonia or lung abscess. Many of the age 50+ patients also exhibited cardiac dysrhythmias and mental disorders. There were also more than 12% with respiratory failure and nearly 10% with shock (see Table 5 for syndromes and Table 6 for sub-syndromes).

**Table 6:** Syndromes assigned to synthetic tularemia injects.

Syndrome	% of 221 visits with listed syndrome
Fever	92.76
Gastrointestinal	12.22
Hemorrhagic Illness	10.86
Localized Cutaneous Lesion	.45
Lymphadenitis	1.81
Neurological	12.67
Rash	4.52
Respiratory	79.64
Severe Illness or Death	11.76
Specific Infection	7.24

Of the 203 patients, 107 had from 1 to 28 laboratory orders. The most common test was a blood culture, for nearly 40% of the laboratory tests. The next most common test was respiratory culture and smear at nearly 13% of the tests (see Table 7). There were also some very specific tests for *C. difficile *toxin (11.33%) and Strep Group A (2.63%). Laboratory results were forced to be negative because of the fastidious nature of tularemia [37]; any bacterial cultures were forced to either exhibit the results "no growth" or normal flora.

**Table 7:** Sub-syndromes assigned to synthetic tularemia injects.

Sub-Syndrome	% of 221 visits with listed Sub-Syndrome	Sub-Syndrome	% of 221 visits with listed Sub-Syndrome
Abdominal Pain	8.14	Heart disease, ischemic	2.26
Alteration of Consciousness	12.67	Hempotysis	7.24
Anemia	10.86	Hypotension	1.81
Asthma	3.62	Influenza-like Illness	8.14
Bronchitis and Bronchiolitis	11.31	Intestinal infections, ill-defined	.45
Cardiac dysrythmias	14.48	Lymphadenopathy	1.81
Chest pain	9.95	Malaise and fatigue	.45
Coagulation defects	.45	Mental disorders	10.86
Coma	8.14	Migraine	.45
COPD	1.36	Nausea and vomiting	9.95
Cough	44.34	Pleurisy	.45
Cyanosis and hypoxemia	2.71	Pneumonia and lung abscess	19.00
Death	.45	Pupurae and petechiae	1.36
Dehydration	1.81	Rash	10.41
Diabetes mellitus	1.81	Respiratory failure	12.67
Diarrhea	8.60	Septicemia and bacteremia	4.52
Dizziness	.45	Shock	9.95
Dyspnea	39.82	Skin infection	.45
Edema	2.71	Syncope and collapse	.45
Fever	47.51	Upper respiratory infections	2.26
Gastrointestinal hemorrhage	2.71	Urinary tract infections	1.81
Headache	.90	Viral infection, unspecified	11.31

Of the 112 synthetic patients who had radiology orders, over 80% had chest x-rays (see Table 8). Radiology results were imposed on the synthetic victims to indicate evidence of bilateral peribronchial infiltrates and multi-lobar opacities, bilateral pleural effusions, bilateral sub-segmental infiltrates with foci of peribronchial consolidation, or miliary patterns of multiple acenodular opacities 3-6 mm in diameter, all characteristic of primary pneumonic tularemia (see Table 9).

**Table 8:** Laboratory Tests for Synthetic Tularemia Injects.

Ordered Test Name Local (Laboratory Test)	Percent of Tests Ordered	Ordered Test Name Local (Laboratory Test)	Percent of Tests Ordered
ASO Titer(ASO)	.66	Influenza Antigen(FLUAG)	3.45
Aerobic	.16	Legionella Ag Urine Culture(LEGEIA)	.82
Blood Culture (BLC)	39.90	Lyme IgG	.66
Blood Culture Isolator(BLDC)	.66	Mono Test (MONO)	.16
C Difficile Toxin A	11.33	Ova and Parasite Exam (OVAP)	2.46
C Reactive Protein (CRP)	3.28	Prealbumin (PAB)	1.64
CMV AB IgG (CMVGAB)	.66	Reproductive Culture	.66
CMV AB IgM(CMVMAB)	.66	Respiratory Culture and Smear(RTCS)	12.97
Chlamydia/GC by Amplified	.66	Respiratory Viral Panel Acute(RVPA)	.66
Probe(CGPT)			
Enteric Pathogen Culture(ENPC)	2.46	Strep Group A Screen Rapid(RSTREP)	2.63
Epstein Barr Virus Antibody	.66	Urine Culture (URC)	10.67
Screen (EBVSRN)			
Gram Smear (GRAS)	.16	Urine Culture and Smear (URCS)	.16
Haptoglobin (HAPT)	1.15		
Herpes Virus Six Culture(HHV6Q)	.66		

There were 609 tests ordered.

**Table 9:** Radiology Orders for synthetic tularemia injects.

Ordered Test Name Local (Radiology Order)	Percent of Tests Ordered
DX Abdomen 2 View	.64
DX Abdomen AP	13.74
DX Abdomen Acute	.64
DX Chest 1 View AP	34.50
DX Chest 1 View NR	1.60
DX Chest 2 View	23.64
DX Chest Special Vi	32
DX Small Bowel Series	.64
PX Abdomen Portable	2.88
PX Chest 1 V Portable	20.77
PX Cholangiogram In	.64

For illustrative purposes, we will now follow two of the synthetic tularemia patients through the various records of the EMR, as seen in Table 10. The first patient, patient ID 214973, analysis visit ID 536919, sought care for prodrome symptoms. We see an analysis visit date of Aug 3 2006, at which the patient reported fever and other flu-like symptoms. The syndromes assigned were fever and respiratory, and the sub-syndromes were fever and influenza-like illness. This patient had one clinical activity record for the chief complaint, no laboratory or radiology orders (or results) and was discharged the same day. The second synthetic tularemia patient, patient ID 210042, analysis visit ID 536120, was an outpatient. This patient had a reason for visit of *coug *(*sic*) and fever and was assigned syndromes of respiratory and fever, sub-syndromes of cough and fever. This patient had four clinical activity records: for chief complaint, working diagnosis and final diagnosis. The patient had one radiology order and three radiology results records. This synthetic patient had a routine discharge.

**Table 10:** Electronic medical records for two synthetic tularemia inject patients.

A) Analysis Visit Table					
Analysis Visit ID	Patient ID	Analysis Visit Date	Analysis Visit End Date	AVPatClass	
**536919**	214973	03AUG2006:05:27:01	03AUG 2006:05:7:01	E	
**536120**	210042	18AUG2006:07:49:21	27AUG2006:18:00:33	O	

( Analysis Visit Table continued...)					

**AVBirthdate**	**AVStandardAge**	**AVAgeRange**	**AVGender**	**AVRaceCode**	**AVRace**

**25AUG1981:00:00:00**	25	20-49	Male	2106-3	White
**03JUN1957:00:00:00**	47	20-49	Female	2106-3	White

(Analysis Visit Table continued...)					

**AVEthnicGroupCode**	**AVEthnicGroup**	**AVDischarge Disp Code**	**AVDischargeDisp**		

**2186-5**	Not Hispanic or Latino	1	Discharged to home or self care (routine discharge)		
**2186-5**	Not Hispanic or Latino	1	Discharged to home or self care (routine discharge)		

(Analysis Visit Table continued...)					

**AVDischargeDate**	**AVPOC**	**AVPOCDate**	**AVDeceaseFlag**	**AVAdmissionTypeCode**	

**04AUG2006:05:37:50**	ER	03AUG2006:05:27:01		E	
**20AUG2006:01:24:37**	RA3	19AUG2006:02:41:11		E	

(Analysis Visit Table continued...)					

**AVCASummary**	**AVBucketSummary**	**AVSubSynSummary**	**AVSynSummary**		

**FEVER,OTHER FLULIKE SYMPTOMS;**	|Emergency - Chief Complaint|	|Fever |Influenza-like illness|	|Fever|Respiratory		
**780.6 FEVER; 786.2 COUGH;COUG AND FEVER**	|Outpatient - Final Diagnosis|Outpatient - Reason for Visit|Outpatient - Working Diagnosis	|Cough|Fever|	|Respiratory|Fever|		

B) Clinical Activity Table					

**AnalysisVisitID**	**PatientID**	**AnalysisVIsitDate**	**PatientClass**	**ActivityType**	**ActivitySource**

**536919**	214973	03AUG2006:05:27:01	E	CC	PV2
**536120**	210042	18AUG2006:07:49:21	O	CC	PV2
**536120**	210042	18AUG2006:07:49:21	O	DX	DG1
**536120**	210042	18AUG2006:07:49:21	O	DX	DG1
**536120**	210042	18AUG2006:07:49:21	O	DX	DG1
(Clinical Activity Table continued...)					

**ActivityCode**	**ActivityText**	**ActivityDate**	**ActivityStatus**	**SourceType**	

	FEVER OTHER FLU LIKE SYMPTOMS	03AUG2006:05:27:01		Hospital	
	COUG AND FEVER	18AUG2006:07:49:21		Hospital	
**786.2**	786.2 COUGH	18AUG2006:07:49:21	F	Hospital	
**780.6**	780.6 FEVER	18AUG2006:07:49:21	F	Hospital	
**786.2**	786.2 COUGH	18AUG2006:07:49:21	A	Hospital	

(Clinical Activity Table continued...)					

**CategoryType**	**EffectiveBeginDate**	**EffectiveEndDate**	**AnalysisAge**	**AnalysisAgeUnit**	

**Chief Complaint**	03AUG2006:06:18:51	03AUG2006:05:27:01	25	Year	
**Reason For Visit**	18AUG2006:22:04:34	18AUG2006:07:49:23	47	Year	
**Final Diagnosis**	27AUG2006:10:31:30	27AUG2006:03:32:34	47	Year	
**Final Diagnosis**	27AUG2006:19:47:07	18AUG2006:07:49:21	47	Year	
**Final Diagnosis**	27AUG2006:17:57:09	18AUG2006:07:49:21	47	Year	

(Clinical Activity Table continued...)					

**AnalysisGender**	**SubSynBitmapText**	**SynBitmapText**	**SubSynIncBitmapText**		

**Male**	|Fever|Influenza-like Illness|	|Fever|Respiratory|	|Fever|Influenza-like Illness|		
**Female**	|Cough|Fever|	|Respiratory|Fever|	|Cough|Fever|		
**Female**	|Cough|	|Respiratory|	|Cough||		
**Female**	|Fever|	|Fever|	|Fever|		
**Female**	|Respiratory|	|Respiratory|	|Cough|		

(Clinical Activity Table continued...)					

**SubSynIncBitmapRules**		**SynIncBitmapText**		**SynIncBitmapRules**	
**|566|4749|**		|Fever|Respiratory|		|2324|3309|	
**|296|566|**		|Respiratory|Fever|		|3298|2324|	
**|2165|**		|Respiratory|		|2885|	

C) There are no laboratory orders for these two patients.					
D There are no laboratory results for these two patients.					
E) Radiology Order Table					

**PatientID**	**AnalysisVisitID**	**OrderControl**	**AnalysisVisitDate**	**AVServicFacType**	

**210042**	536120	NW	18AUG2006:07:49:21	RT	

(Radiology Orders continued...)					

**PatientClass**	**Order Number**	**DiagnosticService**	**ReportDate**	**Results Status**	

**O**	44404327	RAD	19AUG2006:03:01:01	P	

(Radiology Orders continued...)					

**BeginDate**	**OrderedTestCodeLocal**	**Ordered TestNameLocal**	**ReasonForTest**		

**19AUG2006:03:01:01**	15488699	DX Chest 2 View	COUGH AND FEVER		

F) Radiology Results Table					

**PatientID**	**AnalysisVisitID**	**RadResultKeyID**	**ResultHeaderNumber**	**AnalysisVisitDate**	

**210042**	536120		1	18AUG2006:07:49:21	
**210042**	536120		1	18AUG2006:07:49:21	
**210042**	536120		1	18AUG2006:07:49:21	

(Radiology Results continued...)					

**AVServiceFacType**	**PatientClass**	**Order Number**	**Diagnostic Service**	**ReportDate**	**Results Status**

**RT**	O	444004327	RAD	19AUG2006:06:06:32	P
**RT**	O	444004327	RAD	19AUG2006:06:55:41	F
**RT**	O	444004327	RAD	19AUG2006:08:59:32	F

(Radiology Results continued...)					

**ProcedureDate**	**OrderedTestCodeLocal**	**OrderedTestNameLocal**	**ReasonForTest**		

**18AUG2006:14:01:40**	15488699	DX Chest 2 View	COUGH AND FEVER		
**18AUG2006:10:32:15**	15488699	DX Chest 2 View	COUGH AND FEVER		
**18AUG2006:11:15:24**	15488699	DX Chest 2 View	COUGH AND FEVER		

(RadiologyResults continued...)					

Some fields have been omitted for clarity; most of the omitted fields are blank.

Impressions

PA AND LATERAL VIEWS OF CHEST Findings: Multiple parenchymal infiltrates seen bilaterally. There are bilateral pleural effusions

PA AND LATERAL VIEWS OF CHEST Findings: Multiple parenchymal infiltrates seen bilaterally. There are bilateral pleural effusions

PA AND LATERAL VIEWS OF CHEST Findings: Multiple parenchymal infiltrates seen bilaterally. There are bilateral pleural effusions

### III.b. Background Patient Generation

We generated approximately 3000 synthetic background patient EMRs for the 4-11 year age-group. These were generated several times to insure that the algorithms performed consistently. There were 295 different primary diagnosis ICD-9 codes for the group of synthetic emergency patients and 294 different primary diagnosis ICD-9 codes for the group of synthetic outpatients.

These ICD-9 codes included both illness and injury. In fact, the most common primary diagnosis for the emergency room patients in this age group was head laceration. To illustrate, we will follow the two synthetic background patient EMRs described in Table 11. The first (Patient ID 60343) is a 4-year old girl with chief complaint of sore throat. The synthetic patient record has a syndrome assignment of respiratory and sub-syndrome assignment of upper respiratory infections. The clinical activity record has 3 entries - for chief complaint, working diagnosis of pharyngitis, and final diagnosis of pharyngitis. There is one laboratory order and it is for a rapid strep test. There are no laboratory results for rapid strep tests in any of the synthetic EMRs, reflecting the pattern found in the real EMRs. There are no radiology orders or results for this synthetic patient, who had a routine discharge.

**Table 11:** Electronic medical records for two synthetic background patients.

A) Analysis Visit Table				
**AnalysisVisitID**	**Patient ID**	**Visit Count**	**AVFirstVisitID**	**AnalysisVisitDate**

**72835**	60343	1	72835	15JUL2006:18:46:59
**5326**	62834	1	75326	23JUL2007:19:26:24

(Analysis Visit Table continued...)				

**AnalysisVisitEndDate**	**AVPatClass**	**AVBirthdate**	**AVAge**	**AVAgeRange**

**15JUL2006:21:05:04**	E	23JUN2002:00:00:00	4	4-11 Years
**23AUG2007:18:49:19**	E	12SEP1995:00:00:00	11	4-11 Years

(Analysis Visit Table continued...)				

**AVGender**	**AVRaceCode**	**AVRace**	**AVEthnicGroupCode**	**AVEthnicGroup**

**F**	2106-3	White	2186-5	Not Hispanic or Latino
**M**	2106-3	White	2186-5	Not Hispanic or Latino

(Analysis Visit Table continued...)				

**AVDischargeDispCode**	**AVDischargeDisp**	**AVAdmitDate**		**AVDischargeDate**

**1**	Discharged to home or self care (routine discharge)	15JUL2006:20:14:07		15JUL2006:21:07:21
**1**	Discharged to home or self care (routine discharge)	23AUG2007:16:57:59		23AUG2007:18:03:51

(Analysis Visit Table continued...)				

**AVDeceasedFlag**	**AVDeceasedDate**	**AVCASummary**		

		SORE THROAT;|462 |462 LEFT CLAVICULAR PAIN;|810.02|E884.0|786.59		

(Analysis Visit Table continued...)				

**AVBucketSummary**		**AVSubSynSummary**		**AVSynSummary**

**Emergency - Chief Complaint|Emergency - Final Diagnosis| Emergency - Working Diagnosis**		Upper_respiratory_infections		Respiratory
**Emergency-CheifComplaint|Emergency - Final Diagnosis| Emergency - Final Diagnosis| Emergency - Working Diagnosis**			Falls|Fractures_and_dislocation	

B) Clinical Activity Table				

**AnalysisVisitID**	**CAKeyID**	**PatientID**	**AnalysisVisitDate**	**PatientClass**

**72835**	32096443	60343	15JUL2006:18:46:5	E
**72835**	32096444	60343	15JUL2006:18:46:59	E
**72835**	32096445	60343	15JUL2006:18:46:59	E
**75326**	32106099	62834	23JUL2007:19:26:24	E
**75326**	32106100	62834	23JUL2007:19:26:24	E
**75326**	32106101	62834	23JUL2007:19:26:24	E
**75326**	32106102	62834	23JUL2007:19:26:24	E

(Clinical Activity Table continued...)				

**ActivityType**	**ActivitySource**	**ActivityCode**	**Activity Text**	

**CC**	PV2		SORE THROAT;	
**DX**	DG1	462	462 ACUTE PHARYNGITIS	
**DX**	DG1	462	462 ACUTE PHARYNGITIS	
**CC**	PV2		LEFT CLAVICULAR PAIN	
**DX**	DG1	810.02	810.02 FX CLAVICLE SHAFT-CLOSED	
**DX**	DG1	E884.0	E884.0 FALL FROM PLAYGROUND EQUIPMENT	
**DX**	DG1	786.59	786.59 CHEST PAIN NEC	

(Clinical Activity Table continued...)				

**ActivityDate**	**CategoryType**	**AnalysisAge**	**AnalysisAgeUnit**	**AnalysisGender**

**16JUL2006:10:04:00**	Chief Complaint	4	Year	F
**16JUL2006:15:24:31**	Final Diagnosis	4	Year	F
**16JUL2006:02:34:42**	Working Diagnosis	4	Year	F
**26JUL2007:08:09:45**	Chief Complaint	11	Year	M
**26JUL2007:13:39:56**	Final Diagnosis	11	Year	M
**26JUL2007:19:26:24**	Final Diagnosis	11	Year	M
**26JUL2007:19:26:24**	Working Diagnosis	11	Year	M

C) Laboratory Orders Table				

**PatientID**	**AnalysisVisitID**	**LabOrderKeyID**	**ResultHeaderNumber**	**OrderControl**

**60343**	72835		1	NW

(Laboratory Orders Table continued...)				

**ServiceFacID**	**VisitID**	**AnalysisVisitDate**	**AVServiceFacType**	**PatientClass**

**45**	609251	15JUL2006:00:00	RT	E

(Laboratory Orders Table continued...)				

**OrderNumber**	**FillerOrderNumber**	**DiagnosticService**	**OrderDate**	**BeginDate**

**440026009**		MB		15JUL2006:21:50:03

(Laboratory Orders Table continued...)				

**OrderedTestCode**	**OrderedTestName**	**OrderedTestCodingSystem**		**OrderedTestCodeLocal**

				STTH

(Laboratory Orders Table continued...)				

**OrderedTestNameLocal**			**SpecimenTypeLocal**	

**Strep Group A Culture (STTH)**			THT	

D) No Laboratory Results for these patients				
E) Radiology Orders Table				

**PatientID**	**AnalysisVisitID**	**RadOrderKeyID**	**ResultHeaderNumber**	**OrderControl**

**62834**	75326		1	NW

(Radiology Orders Table continued...)				

**ServiceFaciD**	**VisitID**	**AnalysisVisitDate**	**AVServiceFacType**	**PatientClass**

**245**	611742	25JUL2007:19:26:24	RT	E

(Radiology Orders Table continued...)				

**Order Number**	**RadiologyNumber**	**DiagnosticService**	**ReportDate**	**ResultsStatus**

**440067632**		RAD	26JUL2007:19:13:30	P

(Radiology Orders Table continued...)				

**OrderDate**	**BeginDate**	**OrderedTestNameLocal**	**ReasonforTest**	

	26JUL2007:21:05:29	DX Clavicle LEFT	Trauma	

F) Radiology Results Table				

**PatientiD**	**AnalysisVisitID**	**RadResultKeyID**	**ResultHeaderNumb**	**OrderControl**

**62834**	75326	5887812	1	RE
**62834**	75326	5887812	1	RE

(Radiology Results Table continued...)				

**ServiceFacID**	**VisitID**	**AnalysisVisitDate**	**AVServiceFacType**	**PatientClass**

**245**	611742	25JUL2007:19:26:24	RT	E
**245**	611742	25JUL2007:19:26:24	RT	E

(Radiology Results Table continued...)				

**OrderNumber**	**RadiologyNumber**	**DiagnosticService**	**ReportDate**	**ResultStatus**

**440067632**		RAD	27JUL2007:05:42:35	F
**440067632**		RAD	26JUL2007:20:41:53	F

(Radiology Results Table continued...)				

**ProcedureDate**	**OrderedTestCodeLocal**	**OrderedTestNameLocal**		**ReasonForTest**

**26JUL2007:19:41:37**	15488740		DX Clavicle LEFT	Trauma
**26JUL2007:20:39:31**	15488740		DX Clavicle LEFT	Trauma

(Radiology Results Table continued...)				

Some fields have been omitted for clarity; most of the omitted fields are blank.

Impressions

CLINICAL HISTORY: Pain following trauma. Technique: Two views were obtained. Comparison: None. Findings: There is a superiorly apex angulated, nondisplaced fracture of the mid clavicle. No pneumothorax is seen based on the view submitted. IMPRESSION: Nondisplaced fracture of the mid clavicle.

CLINICAL HISTORY: Pain following trauma. Technique: Two views were obtained. Comparison: None. Findings: There is a superiorly apex angulated, nondisplaced fracture of the mid clavicle. No pneumothorax is seen based on the view submitted. IMPRESSION: Nondisplaced fracture of the mid clavicle.

The second synthetic patient is an 11-year old boy with a chief complaint of clavicular pain. The analysis visit record has sub-syndromes of falls and fractures and dislocation. There are 4 clinical activity records: for chief complaint, working diagnosis (chest pain) and final diagnosis (closed clavicle fracture and fall from playground equipment). This synthetic patient record has one radiology order, for a clavicle x-ray, and 2 radiology results, reflecting the diagnosis of clavicle fracture. This synthetic patient had a routine discharge.

The descriptors for the synthetic background patients were built from the truncated 3 or 4- digit ICD-9 codes. Thus, we encountered an occasional mismatch between the detailed ICD-9 codes of the synthetic patient and the detailed ICD-9 codes of the closest care model for that patient. These mismatched records accounted for the majority of the errors that were present in the synthetic EMRs before we corrected them. For example, a synthetic patient's final diagnosis ICD-9 for a dog bite was matched to the care model for a non-venomous insect bite. However, these errors were present in fewer than 3% of the synthetic patient records (see Table 12). The remaining 97% of the synthetic patient records (including all tables relating to the patient: laboratory orders and results, radiology orders and results, clinical activity and visit records) exhibited expected care patterns and visit timelines for the synthetic patients.

**Table 12:** Validation of Synthetic background electronic medical records.

Error	Number of Errors found	Percent of total Records
Care Model did not match ICD-9	80	2.4%
codes completely.		
ICD-9 Codes contradictory	8	.24%
Gender or Age ICD-9 mismatch	6	.18%
Sub-syndrome or syndrome	4	.12%
assignment inconsistent with ICD-		
9 or chief complaint		
Typographical or formatting error	4	.12%
Total	91	2.8%

There were 3272 visit records.

Overall, there were few errors in the synthetic background patients. As we see from Table 12, other than the rarely mismatched care models, errors could occur in the synthetic visit records themselves. There were errors concerning ICD-9 codes that were either in the wrong season (e.g. frostbite in the summer) or ICD-9 codes that should not appear together during the same visit for the same patient (e.g. a code for nausea and vomiting in addition to a code for vomiting alone). There were some syndrome and sub-syndrome assignments that seemed strange and for which examples could not be found in the real data. There were also occasional typographic or formatting errors that were inserted by the algorithms rather than present in the original text. However, overall, fewer than 3% of the records exhibited either obvious or non-obvious errors after inspection by a subject matter expert.

**Figure 10:**
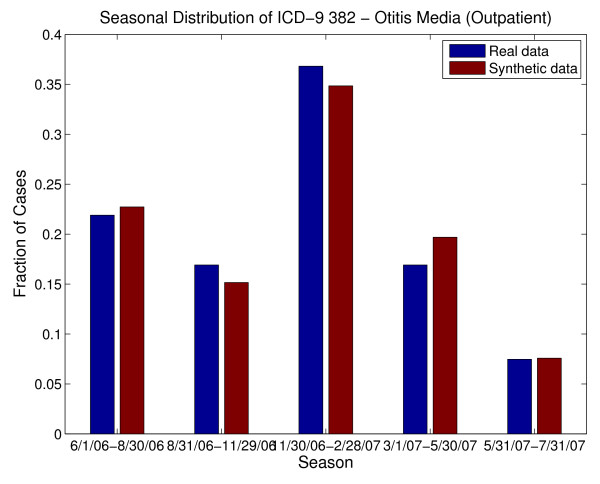
**Original and synthetic seasonal distribution for ICD-9 code 382 (Otitis Media) - outpatient cases. This ICD-9 accounted for more than 7% of all outpatient visits**.

**Figure 11:**
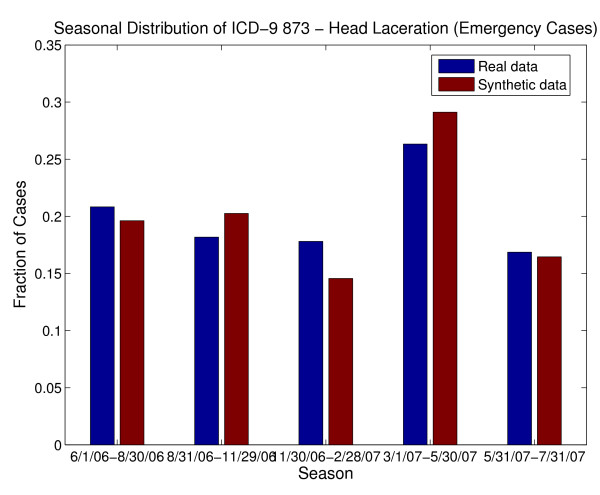
**Original and synthetic seasonal distribution for ICD-9 code 873 (head laceration) - emergency cases. This ICD-9 accounted for more than 9% of all emergency visits**.

We discussed the reproduction of various statistical and temporal patterns in the synthetic data in [49]. Overall, the synthetic records were able to mimic most statistical attributes of the real data quite well. In addition, seasonal and day-of-week characteristics of the real data were reproduced in the synthetic data. To illustrate, consider Figures 10 and 11 showing the seasonal distributions for Otitis Media in outpatient cases and Head Lacerations in Emergency cases, respectively. In this age group, Otitis Media cases accounted for over 7% of all outpatient visits and head lacerations accounted for more than 9% of emergency visits. We see from these figures that the seasonal variation in both of these were reproduced well in the synthetic data.

The complete synthetic tularemia and background data sets can be found on the public health grid [11] once acceptable security controls are in place.

## IV. Discussion and Conclusions

Due to privacy concerns, even sanitized and anonymized EMRs cannot be shared among researchers developing bio-surveillance algorithms, methods for improving the quality of patients' care or investigating adverse drug effects. The work presented in this paper aims at removing this obstacle (i.e., the lack of non-identifiable EMR data). These new areas of research can only thrive when abundant, shareable and complete EMR data are available for use.

We have developed a data-driven method for generating full synthetic EMRs of tularemia patients as well as of background patients. The method has three major steps: 1) synthetic patient identity and basic information generation; 2) identification of care patterns that the synthetic patients would receive; and 3) adaptation of patient care models. The techniques described herein s are data-driven, meaning that these techniques mine the data in existing real EMRs in order to extract information about the patients' patterns of care, the frequencies of ICD-9 codes, syndromes, and sub-syndromes. The synthetic EMRs need to mimic rather than duplicate the real EMRs. That is, no synthetic patient can match a real patient exactly in age, gender, demographic variables and visit information, although as a group the age, gender, demographic variables and diagnoses need to display the same statistical distributions as the real EMRs.

In the case of tularemia inject generation, 203 synthetic victim records were synthesized. 19 victims sought care for the prodrome, and all the victims sought care for the full blown illness. Examples of full EMRs of two synthetic patients were described in detail. The method developed lends itself to generating EMRs of patients with illnesses other than tularemia. If such illnesses are present in the data set, the methodology will be the same as the one developed for the synthetic background data. For illnesses not present in the data set (e.g., those which are bioterrorism related), the illness needs to be studied using case reports and other information found in the medical literature. Also, expert medical opinion needs to be taken into consideration (similar to what we present here for tularemia) in order to find patterns of care in the data that match the sequence of symptoms that will be generated in the course of modeling the illness's progression in each synthetic victim. For each new illness not present in the data, this may be a time consuming process.

For the most part, patients 4-11 years old become sick or injured, get treated, and get well. For this reason, the 4-11 age group was the least complicated with which to develop the synthesis algorithms. Although there are quite a few patients in this age group with chronic conditions such as asthma, there were few with co-morbid conditions that cause repeated visits and extended hospital stays. Thus the visits are rarely related one to another, which allowed us to develop a methodology that performs matching on visits, instead of matching on the full Patient Care Model.

The pilot synthetic background data set was a starting point for the evolution of ideas; other methods will be necessary to synthesize patients in other age groups. It will be necessary to separate patients into categories of one-time visits that concern one or a few transient illnesses or injuries, and multiple related visits with dependent causes and co-morbid conditions. The methodology of matching Visit Care Models that we developed for the pilot age group will be insufficient to create reasonable synthetic records for the older groups of patients (especially 50+). For these patients, Patient Care Models will need to be used for matching instead of Visit Care Models. Thus the methodology for the older age groups will resemble the method developed for synthetic injects in which the matching was done on full Patient Care Models. We will need to develop care models for coexistent illnesses (e.g., diabetes and hypertension) that vary in manifestations of both or either underlying illness. In this case, the publications based on the Archimedes model [21] will be helpful in describing possible manifestations of illnesses. These can assist in the development of care patterns that are consistent with the format and protocols present in the particular real data that are used for a model.

We highlight the usefulness of this method with regard to the injection of electronic medical records of victims of bioterrorism or naturally occurring outbreaks of infectious disease. Our three-step method of producing visit records of the synthetic victims, matching them to the closest model of care, and adapting the closest model of care to the specific disease (or injury) can be used to test and develop many classes of algorithms and monitoring systems that operate on the entire electronic medical record.

## Competing interests

The authors declare that they have no competing interests.

## Authors' contributions

ALB created the concepts of patient care model, patient care descriptor, and analysis visit descriptor, as well as the algorithms for creation of all the descriptors. She defined the distance measures and run the algorithms for retrieving the closest ten descriptors. She co-authored the manuscript.

SB served as a medical expert on the creation of synthetic tularemia records and assisted in the validation of the synthetic tularemia records. He performed the validation of the synthetic background records and assisted in the editing of the manuscript.

LM was the project's technical lead. She created the algorithms and executed the majority of the programs for creation of synthetic patient identities for both injected tularemia patients and synthetic background patients. She wrote and executed the care model adaptation and injection algorithms. She performed validation of statistical properties of the records. She co-authored the manuscript.

All authors read and approved the final manuscript.

## Pre-publication history

The pre-publication history for this paper can be accessed here:

http://www.biomedcentral.com/1472-6947/10/59/prepub
